# Are We Helping Workers Reskill for the Future of Work? Using AI to Explore the Alignment of Online Course Offerings and Job Skill Requirements

**DOI:** 10.3390/jintelligence14040059

**Published:** 2026-04-01

**Authors:** Makai A. Ruffin, Margaret E. Beier, Felix Y. Wu, Nathaniel M. Voss, Anoop A. Javalagi, Harrison J. Kell

**Affiliations:** 1 Department of Psychological Sciences, Rice University, Houston, TX 77005, USA; 2 Human Resources Research Organization (HumRRO), Alexandria, VA 22314, USA

**Keywords:** talent development, artificial intelligence, reskilling, future of work

## Abstract

Millions of workers and job seekers turn to online platforms to gain work-relevant skills to remain competitive for the future of work. However, little is known about whether the skills acquired in work-relevant online courses align with the skills required for 21st-century jobs. Drawing on literature on job and skill matching, this exploratory study examines the alignment between available online training and learning content and the skills demanded by jobs (i.e., training-skills demands fit) using artificial intelligence methods. A large language model (LLM; Claude Haiku 3.5) was instructed to evaluate which of the 35 basic and cross-functional skills from the Occupational Information Network (O*NET) could be acquired in a given course, which was based on 2549 course descriptions extracted from MIT OpenCourseWare. Linkages between online training and skills were broken down by job family and occupations with a bright outlook designation (i.e., occupations estimated to have 75,000 or more job openings between 2024 and 2034 across the United States). Results suggest that the skill of active learning (i.e., using new information for problem-solving; 88%, *N* = 2242) was linked to the highest number of online courses, whereas the skill of instructing (i.e., teaching others to perform tasks; 5.3%, *N* = 134) was linked to the least. Computer and mathematical occupations had the highest proportion of courses wherein individuals can acquire basic and cross-functional skills, whereas food preparation and serving occupations had the lowest proportion of courses. Non-bright outlook occupations had a significantly lower proportion of online courses where individuals can acquire basic and cross-functional skills compared to occupations with a bright outlook designation. We expand on existing skills-matching perspectives to consider how training-skills demands fit can constrain or facilitate continuous learning and development. Further, we illustrate how LLMs can be used to efficiently and at scale summarize descriptive information on talent development issues.

## 1. Introduction

Rapid advances in automation and artificial intelligence (AI) may require up to 375 million workers worldwide to change occupations to stay competitive (Manyika et al., 2017). Expressed differently, it is estimated that close to 12 million occupations will require transitions due to technological advancements (Hazan et al., 2024). To meet this challenge, workers and organizations are investing resources (e.g., paid time off, tuition reimbursements, coverage of course fees; Castaño Muñoz et al., 2016) in work-relevant self-directed learning activities (i.e., learning that occurs both within and outside organizations, focused on acquiring work knowledge or skills; Beier et al., 2025) to reskill. Reskilling refers to training and development activities designed to help workers and job seekers gain new skills to be qualified for a new role or enter new professions (Cole, 2018). We define skills as “proficiencies that are developed through training or experience” (Fleisher & Tsacoumis, 2012, p. 1). In other words, skills refer to what a person can currently do (Campbell, 1990; Sackett et al., 2017). People are increasingly engaging with online platforms and tools (e.g., massive open online courses, open-source course materials) to close the gap between their current and desired skills. Indeed, approximately 20% of learners used online learning platforms to build new knowledge and skills to switch to a different job or career field (Coursera, 2025). Investment in reskilling activities is also beneficial for organizations, as it allows them to stay relevant in an evolving labor landscape and retain talent through investment in skill development (Ruíz-Valdés et al., 2023). Consequently, organizations are increasingly pivoting toward skills-based hiring models, prioritizing demonstrated competencies over traditional credentials to widen their talent pools (Agovino, 2024; VanDerziel et al., 2025).

The assessment of skills is crucial to talent development, and prior research has attempted to predict the skills most important to remain employed and employable for the future of work. For instance, Li (2024) delineated the skills relevant for Industry 4.0 (i.e., a process of revolutionizing manufacturing and engineering globally) in 2015, 2020, and 2025. In 2015 and 2020, the top skill employees needed to thrive in Industry 4.0 was complex problem-solving; however, in 2025, this dropped two places and was replaced with analytical thinking and innovation skills. Other skills that are projected to grow in importance are technological skills (e.g., AI, big data, cybersecurity), lifelong learning, leadership and social influence, and talent management (Leopold, 2025). Further, to continually acquire new skills, additional socio-emotional skills, such as flexibility and curiosity, are necessary for people to remain competitive in the labor market (OECD, 2019).

Researchers are striving to identify the skills necessary for the future of work; however, 39% employers expect key skills to change in demand (Leopold, 2025). To address these expectations, millions of people engage in online learning platforms to gain in-demand skills. However, it remains to be seen (1) what in-demand skills are advertised on online learning platforms, and (2) whether skills advertised on online learning platforms align with skills required for many occupations. Drawing on perspectives from psychology, economics, and organizational science, we adopt an interdisciplinary approach to explore *training-skills demands fit*, which we define as the extent to which courses provided on online learning platforms align with skills in demand in the modern workplace. An interdisciplinary approach allows us to integrate the theoretical strengths of psychology, economics, and organizational science with the analytic strength of the emerging field of AI to investigate work-relevant learning in an ecologically valid context.

The current study has several theoretical, methodological, and practical contributions. First, from a theoretical standpoint, the current study highlights the importance of learning ecosystems that facilitate or impede lifespan development (Bronfenbrenner, 2009). Typical theoretical models of self-directed learning focus on individual learner characteristics (e.g., cognitive ability, self-regulation, self-efficacy; micro-level factors) and the learner’s immediate environment (e.g., classroom, technology-assisted learning, and organizational climate for learning; meso-level factors; Garrison, 1997), but rarely incorporate larger contextual factors, such as the availability of learning opportunities, on the macro-level (Lyndgaard et al., 2024). The consideration of macro-level influences on learning is important because these influences reflect overarching societal and economic systems that indirectly shape individual learning and development (Bronfenbrenner, 2009). In this paper, we broaden consideration of the learning ecosystem to include the alignment of skills trained in available learning programs with in-demand skills. After all, whether or not a person has access to skill-relevant learning represents an important boundary condition to their success in the modern workplace.

From a methodological and practical standpoint, we leverage AI methodologies as a viable approach to explore relationships between occupational and learning environment data at scale. In doing so, we add to the growing body of literature using machine learning (ML), large language models (LLMs), and generative AI tools to address organizational and educational issues. Finally, by assessing the extent to which training-skills demands are met, the current study may help workers and job seekers make informed decisions regarding investment and participation in online courses for reskilling activities. With better-informed decisions on skill development activities, workers and job seekers gain cognitive, technical, and socioemotional skills that keep them competitive for the future of work, and organizations are staffed with a proficient workforce.

## 2. Literature Review

### 2.1. Job and Skills Matching

Job matching is a concept often used in the field of economics, which refers to “the alignment of an individual’s skills, interests, and values with the requirements of a position, company culture, and industry trends in order to achieve the optimal combination between the individual and the job” (Gao & Feng, 2024, p. 2). According to job matching literature, workers are not equally suited for all positions, and this variety of talent results in job-specific differences in productivity (Barron et al., 1989; Topel, 1986). A misalignment between an individual’s skills, interests, and values and the requirements of a position, company culture, or industry trends can result in negative individual-level outcomes (e.g., reduced earnings, increased unemployment; Rudakov et al., 2022; Salahuddin et al., 2023; Serikbayeva & Abdulla, 2022). A misalignment can also affect organizational-level outcomes, such as reduced employer hiring preferences (Fu et al., 2021) and on-the-job training requirements and opportunities (Barron et al., 1989; Korpi & Tåhlin, 2021). Alternatively, when there is a high-quality match, there are increases in individual-level (e.g., performance, job satisfaction; Kristof-Brown et al., 2005) and organizational-level (e.g., human capital resources; Ployhart et al., 2014) benefits. One recent extension of job matching is skills matching, which is the alignment between individuals’ skillsets and organizations’ skill needs (Jooss et al., 2024). Discussion around skills matching in talent development literature is less emphasized (Jooss et al., 2024); however, matching skills with organizations’ needs addresses how organizations can improve their alignment with human capital and the extent to which workers fit in dynamic work environments (Chalutz-Ben Gal, 2023).

We explore training-skills demands fit, which focuses on the alignment between the skills taught in online learning platforms and the skills required for employment in the 21st-century workplace. Although job and skills matching literature focuses on the individual worker’s match with their job, we believe the logic behind it extends to macro-level questions about the alignment between the skills trained in online platforms to those relevant in the modern workplace. Macro-level trends highlight the affordances of the ecosystems in which individual learners operate (Bronfenbrenner, 2009). If the training environment does not offer the necessary skills, it will be harder for learners to be successful.

### 2.2. O*NET and the O*NET Content Model

The Occupational Information Network (O*NET) is a publicly available database containing approximately 900 occupation profiles and over 55,000 jobs across the United States (U.S.) economy (National Center for O*NET Development, 2025a). Sponsored by the U.S. Department of Labor, the O*NET uses multi-method data collection procedures (e.g., employer job postings, job incumbents, ML) to provide a comprehensive overview of worker attributes and job characteristics (National Center for O*NET Development, 2025a; Peterson et al., 2001).

The O*NET content model is a useful framework for identifying information about occupations and individuals through job- and worker-oriented descriptors (National Center for O*NET Development, 2025b). Occupations are organized into *job families* based on the work performed, skills, education, training, and credential requirements (National Center for O*NET Development, 2025b). Additionally, the database categorizes specific roles within job families as *bright outlook* occupations, which are expected to have a large number of job openings in the next decade (National Center for O*NET Development, 2025c, 2025e).

The model comprises six major domains: worker characteristics, worker requirements, experience requirements, occupational requirements, workforce characteristics, and occupation-specific information (National Center for O*NET Development, 2025b). Worker characteristics include enduring characteristics, such as worker abilities, general occupational interests, work values, and work styles. Worker requirements are descriptors of acquired attributes that an individual developed through experience and education. Within this domain, skills are further divided into developed capacities that facilitate (1) learning or the more rapid acquisition of knowledge (i.e., basic skills) and (2) performance of activities that occur across jobs (i.e., cross-functional skills; National Center for O*NET Development, 2025b). Experience requirements are related to the experiential background of workers in a particular occupation, including licensing, certification, and training requirements. Occupational requirements describe information about contextual variables (e.g., organizational- and work-related) and work activities, ranging from generalized (i.e., activities common across a large number of occupations), intermediate (i.e., activities that are common across many occupations), to detailed (i.e., specific activities performed across a small to moderate number of occupations). Workforce characteristics include details about global contextual variables, such as current labor market information and projections of future economic conditions for a given occupation. Finally, occupation-specific information includes details ranging from titles and codes used to identify occupations, alternate job titles, and tools critical to the performance of a specific occupation.

Prior research has used the O*NET content model to support the identification and assessment of talent. For instance, regarding issues of fitting new labor market entrants to appropriate and available occupations, Liu et al. (2025) evaluated an integrative set of person-occupation fit assessments that measure fit across vocational interests, work values, knowledge, skills, and personality. Using occupational variables from the O*NET, they found evidence that integrating across the five fit domains led to improvements in model predictions for work-relevant outcomes, such as career choice and career success (both subjective and objective). Moreover, prior research has used O*NET data to address questions about whether talent should be trained on basic skills in addition to technical or niche skill training. Findings suggest that time spent developing talent on basic skills (e.g., basic literacy, social interaction) is foundational to whether the benefits of more specialized skills (e.g., analysis, manual dexterity, reasoning, static strength) are realized (Lee et al., 2025). As such, information from O*NET is intended to help answer broad talent development questions, such as what skills should be developed, how to link educational programs to occupational standards, and how to prepare new entrants or re-employ laid-off or disabled workers (Conte & Landy, 2016; Peterson et al., 2001).

### 2.3. Application of Artificial Intelligence Methodologies to Talent Development Issues

AI and ML are powerful tools that actively transform the methods and analysis researchers and practitioners use to sift through data. These tools continue to evolve, and researchers across several fields have used them to address talent development issues more broadly. For example, Coraggio et al. (2025) used ML techniques on employer-employee data to investigate the correlation between job mismatch, wage, turnover, and firm performance. Results suggest that when workers are better matched to their jobs, they earn significantly more throughout their careers, are less likely to switch to a new employer, and firms experience increased productivity. In the context of recruitment, Dossche et al. (2026) used ML methods to predict which online job vacancies are likely to suffer from prolonged durations (i.e., hard-to-fill roles). Vacancies were easier to fill when there were adjustments to skill requirements and the type of contract (i.e., temporary versus part-time positions), which has implications for how employers attract and recruit high-quality personnel.

Furthermore, recent research has used AI and ML tools in conjunction with the O*NET to examine talent development issues across the employee lifecycle. Nieves et al. (2024) leveraged employment data from the Bureau of Labor Statistics and data on skills and work activities from the O*NET to identify skill gaps between payroll and timekeeping clerks. Using an AI algorithm, they identified skill gaps across three categories for payroll and timekeeping clerks: resource management (e.g., management of financial and material resources), technical (e.g., operation monitoring), and social (e.g., coordination, negotiation). Further, Xu et al. (2023) used a ML framework best suited for natural language processing (NLP) techniques to analyze task statements from the O*NET to predict the automatability of tasks within the next decade. Results suggest approximately 25.1% of occupations within the O*NET are at substantial risk of automation, with specific industries more at risk than others (e.g., accommodation and food services, administrative and support services, manufacturing). Moreover, researchers have applied an algorithm to identify missing skills that may hinder workers from easily transitioning between occupations using O*NET data. Adopting a case study approach on the Washington, D.C. regional economy, when identifying skills required to make the transition between restaurant servers and computer user support specialists, skills that were more likely to hinder a transition between these two occupations were creative thinking and complex problem-solving, both of which would require more emphasis in a retraining program (Waters & Shutters, 2022).

**Figure 1:**
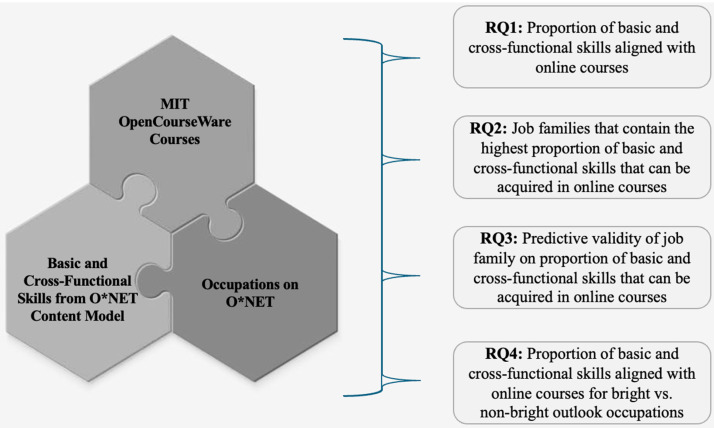
Relationships between courses, skills, and occupations to research questions. Note: RQ represents research questions.

These studies suggest that researchers can incorporate AI methodologies to derive data-driven frameworks for identifying skill gaps, excesses, and matches. Further, AI methodologies allow researchers to adopt macro-level approaches to issues related to talent development. We contribute to this growing body of literature by utilizing a LLM to investigate the degree to which online course descriptions align with basic and cross-functional skills. Our research questions are the following (see Figure 1):

**Research Question 1**: Which basic and cross-functional skills derived from the O*NET are the most aligned with online courses?

**Research Question 2:** Which job families contain the highest percentage of basic and cross-functional skills that can be acquired in online courses?

**Research Question 3:** Does job family predict the proportion of online courses where basic and cross-functional skills can be acquired?

**Research Question 4:** Do occupations with a bright outlook designation contain a larger proportion of online courses where basic and cross-functional skills can be acquired compared to non-bright outlook occupations?

## 3. Methods

### 3.1. Data Sources

#### 3.1.1. Sourced from MIT OpenCourseWare

**Course Descriptions.** We extracted course descriptions from 2549 courses on MIT OpenCourseWare (Massachusetts Institute of Technology: MIT OpenCourseWare, n.d.), which contains an extensive repository of freely available, online course offerings covering a wide range of topics, making it a critical resource for work-relevant self-directed learning, particularly for those without access to paid corporate learning management systems. Examples of course descriptions are in our Open Science Framework (OSF) repository (Table S1): https://osf.io/eqxfk/overview?view_only=adb48666ad5c435ca0901f1956c087e9 (accessed on 21 March 2026).

#### 3.1.2. Sourced from O*NET

Data sourced from the O*NET was from version 30.0.

**Skills.** Occupational analysts are provided information about an occupation’s title and definition, generalized work activities, work contexts, knowledge domains, and core and supplementary tasks to help them determine skill level and importance ratings (Tsacoumis & Willison, 2010). Skills have been rated on their level and importance on a 5-point Likert scale (1 = *Not important* to 5 = *Extremely important*), resulting in 35 skills grouped in seven categories (see Table 1): content (e.g., reading comprehension, active listening), process (e.g., learning strategies, critical thinking), social (e.g., persuasion, negotiation), complex problem-solving, technical (e.g., troubleshooting, programming), systems (e.g., judgment and decision-making), and resource management (e.g., time management, management of financial resources). A total of 884 occupations were examined, with each occupation containing a rating for all 35 skills. 

**Job Family.** Job families are groups of occupations that share commonalities between the work performed, skills, education, training, and credential requirements (National Center for O*NET Development, 2025d, 2025f). Table 2 lists the job families included in the study along with example occupations.

**Bright Outlook Occupation Designation.** Using data from the Bureau of Labor Statistics employment projections, occupations with a bright outlook designation are expected to experience rapid growth in the next several years. Bright outlook occupations are estimated to have 75,000 or more job openings due to job growth and replacement between 2024 and 2034 across the United States (National Center for O*NET Development, 2025c, 2025e). Examples of bright outlook occupations are electricians, registered nurses, food preparation workers, and childcare workers.

### 3.2. Analytic Procedure for LLM-Assisted Analyses

**Figure 2:**
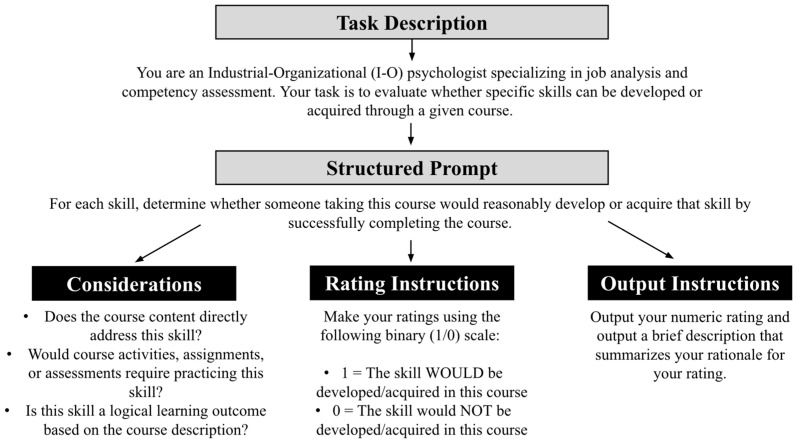
Prompt used for LLM-generated course-skill linkages.

To make the linkages between course descriptions and skills from the O*NET (i.e., course-skill linkages), we prompted an LLM to evaluate whether each of the 35 O*NET skills could be acquired in a given course. We engaged in initial experimentation with the prompt wording and instructions. The final prompt was determined after a specific configuration of instructions produced suitable outputs that were well aligned with the goal of the current study (see Figure 2). Because we examined 2549 course descriptions and 35 skills, a total of 89,215 LLM ratings were made (35 skills × 2549 courses = 89,215 course-skill linkages). To generate numerical ratings, we used Claude Haiku 3.5 (Anthropic, n.d.), as this model strikes a balance between efficiency, strong reasoning capabilities, and costs. As a result, Claude Haiku 3.5 was well-aligned with the goal of this study, which aimed to explore the viability of an LLM for efficiently creating a large number of linkages between online course descriptions and basic and cross-functional skills. For the Claude Haiku 3.5 model, we set both the *top p* and *temperature* hyperparameters, which control the stochasticity of outputs of this LLM, to 0.20, to favor more deterministic (i.e., replicable) ratings and rationales. The LLM was instructed to make a binary judgment about whether a given skill could be acquired in a given course (i.e., 1 = *Skill would be acquired in course*, 0 = *Skill would not be acquired in course*). The LLM was also instructed to provide a rationale for its rating, allowing us to further evaluate the appropriateness of the ratings it generated. We provide example rationales that the LLM provided as supplements to its numeric ratings for one of the courses (“Introduction to Computers and Engineering Problem Solving”) in our supplemental materials on OSF (see Table S2).

### 3.3. LLM Comparisons

We generated a second set of course-skill ratings with a different LLM (Llama 3.3 70B; Meta AI, n.d.) to scrutinize the appropriateness of the ratings generated by the Claude Haiku 3.5 model. The goal of computing a second set of ratings was to examine the consistency of ratings generated across multiple, distinct LLMs, thereby ensuring that ratings are not attributable to model idiosyncrasy. Out of the 89,215 total course-skill linkages, Claude Haiku 3.5 and Llama 3.3 70B provided identical ratings for 85% (*n* = 75,800). The Llama 3.3 70B model made a slightly higher number of course-skill linkages (*n* = 31,241) than the Claude Haiku 3.5 model (*n* = 30,981). This suggests that course-skill linkage ratings are consistent, and the results are not substantially driven by LLM idiosyncrasies. Given that the Claude Haiku 3.5 model was slightly more conservative with its course-skill linkage ratings, coupled with its superior performative capabilities, we report results for the course-skill linkages made with the Claude Haiku 3.5 model.

### 3.4. Human Validation of LLM-Generated Course-Skill Linkages

We assessed the reliability of course-skill linkages made with the Claude Haiku 3.5 model with six subject matter experts (SMEs) with graduate training in Industrial-Organizational Psychology. We used Cochran’s sample size formula to determine the number of course-skill linkages SMEs would need to assess to establish reliability with the course-skill linkages made with the Claude Haiku 3.5 model (Cochran, 1977). Results suggest SMEs rate 384 course-skill linkages; however, SMEs were provided 400 linkages to ensure adequate power. To manage the SME workload, we used a split-panel design where the 400 course-skill pairs were divided into two sets of 200. Each set was then assigned to a subgroup of three independent human raters.

We implemented a unanimous consensus approach to establish a rigorous human ground truth (Reidsma & Carletta, 2008). This approach requires total agreement among the three assigned human raters to verify a linkage. A confusion matrix of the agreement between the LLM and unanimous human rater consensus is in Table 3. The LLM demonstrated strong alignment with this SME baseline, achieving an overall agreement rate of 71.36%. The LLM also demonstrated high sensitivity, such that the model correctly identified skills that were present in course descriptions 82.22% of the time. This indicates that the LLM successfully captures the vast majority of course-skill linkages identified by human raters. Though the LLM was more inclusive than humans in its identification of basic and cross-functional skills present in course descriptions, the results illustrate that the LLM provides a consistent and highly sensitive mechanism for scaling skill extraction. This was especially salient in a domain where coding by human experts is often time-prohibitive and subject to variability in interpretation. R code and output can be found in our repository on OSF.

### 3.5. Analysis of Research Questions

To address Research Question 1, we summed the number of online courses on MIT OpenCourseWare that were linked to basic and cross-functional skills, as well as broader skill categories. To assess which job families contain the highest percentage of basic and cross-functional skills that can be acquired in online courses (i.e., Research Question 2), we extracted data on skill ratings across 884 occupations from the O*NET 30.0 Database (National Center for O*NET Development, 2025g) and estimated the average number of online courses that align with each skill by occupation interaction. The O*NET 30.0 Database classifies each occupation into a job family (National Center for O*NET Development, 2025d, 2025f), allowing estimates on the proportion of online courses by job family.

To address Research Questions 3 and 4, we adopted a linear regression approach for two reasons. First, more sophisticated models (e.g., random forest) generally perform better with larger sample sizes (Cho et al., 2024). Second, given that this work is exploratory in nature, a linear regression approach is an effective baseline model to evaluate whether more complex models and methodologies are worth the additional complexity. To explore whether job family and bright outlook occupations were predictive of the proportion of online courses that cover basic and cross-functional skills described in the O*NET, job family and bright outlook designations were entered as categorical predictors. Similar to how the O*NET 30.0 Database classifies occupations into job families, occupations are also classified into bright versus non-bright outlook occupations (National Center for O*NET Development, 2025c, 2025e). We used protective services as the reference predictor because it served as the median of the average number of online courses that cover basic and cross-functional skills, as reported in the O*NET.

## 4. Results

### 4.1. Descriptives by Skills and Job Family

Table 4 contains the number and percentage of courses from MIT OpenCourseWare that were linked by skill and overall skill category. The skill that could be acquired in the highest percentage of online courses was active learning (i.e., understanding new information for current and future problem-solving and decision-making; 88%, *N* = 2242), followed by critical thinking (i.e., using logic and reasoning to identify strengths, weaknesses, alternative solutions, conclusions, or approaches; 81.5%, *N* = 2078)—both of which are process skills (i.e., procedures that contribute to rapid acquisition of knowledge across domains; Tsacoumis & Willison, 2010). The skill that could be acquired in the lowest percentage of online courses was instructing (i.e., teaching others how to do something; 5.3%, *N* = 134), followed by installation (i.e., installing machines, wiring, equipment or electronic programs; 6.4%, *N* = 162) and operations monitoring (i.e., observing dials and other indicators to ensure machines are working effectively; 7.5%; *N* = 162). Interestingly, the majority of the technical skills were associated with fewer than 20% of courses (8 out of 11 technical skills).

Regarding Research Question 2, we found that on average, computer and mathematical occupations had the highest percentage of basic and cross-functional skills linked to online courses (*M* = 0.45, *SD* = 0.04), closely followed by life, physical, and social science occupations (*M* = 0.44, *SD* = 0.04). Job families such as food preparation and serving (*M* = 0.30, *SD* = 0.07), building, grounds cleaning, maintenance (*M* = 0.30, *SD* = 0.08), and installation, maintenance, and repair (*M* = 0.31, *SD* = 0.05) had the lowest percentage of basic and cross-functional skills linked to online courses on average (see Table 5).

### 4.2. Regression Models

Research Questions 3 and 4 explore whether job family and bright outlook occupations were predictive of the proportion of online courses that cover basic and cross-functional skills described in the O*NET. The overall model was significant (*F*(21, 862) = 27.47, *p* < 0.001) explaining approximately 40% of the variance of the proportion of online courses that cover basic and cross-functional skills ($R^{2}$ = 0.40). Regression coefficients are presented in Table 6. Compared to protective services, architecture and engineering (β = 0.04, *SE* = 0.01, *t* = 3.04, *p* < 0.01), arts, design, entertainment, sports, and media (β = 0.03, *SE* = 0.02, *t* = 1.98, *p* < 0.05), business and financial operations (β = 0.04, *SE* = 0.01, *t* = 2.53, *p* < 0.05), computer and mathematical (β = 0.06, *SE* = 0.02, *t* = 3.76, *p* < 0.001), and life, physical, and social science job families (β = 0.05, *SE* = 0.01, *t* = 3.36, *p* < 0.001) had a significantly higher proportion of online courses wherein individuals can acquire basic and cross-functional skills. Alternatively, compared to protective services, building and grounds cleaning and maintenance (β = −0.09, *SE* = 0.02, *t* = −3.71, *p* < 0.001), construction and extraction (β = −0.06, *SE* = 0.01, *t* = −4.37, *p* < 0.001), food preparation and serving (β = −0.09, *SE* = 0.02, *t* = −4.87, *p* < 0.001), installation, maintenance, and repair (β = −0.08, *SE* = 0.01, *t* = −5.52, *p* < 0.001), personal care and service (β = −0.05, *SE* = 0.02, *t* = −3.09, *p* < 0.01), production (β = −0.07, *SE* = 0.01, *t* = −5.45, *p* < 0.001), and transportation and material moving job families (β = −0.06, *SE* = 0.01, *t* = −4.15, *p* < 0.001) had a significantly lower proportion of online courses wherein individuals can acquire basic and cross-functional skills.

Further, using occupations with a bright outlook designation as the reference group, the overall model was significant (*F*(1, 880) = 10.61, *p* < 0.01), explaining approximately 1.19% of the variance of the proportion of online courses that cover basic and cross-functional skills ($R^{2}$ = 0.01). Regression coefficients are presented in Table 7. Compared to occupations with a bright outlook designation, non-bright outlook occupations had a significantly lower proportion of online courses in which individuals can acquire basic and cross-functional skills (β = −0.02, *SE* = 0.01, *t* = −3.26, *p* < 0.01).

## 5. Discussion

Many individuals turn to online learning platforms to gain work-relevant skills; however, little is known about the extent to which the content shared in online courses aligns with skills necessary for the future of work. The current study took a step to address this gap using an LLM (a specialized type of AI) to assess whether course descriptions from one online learning platform, MIT OpenCourseWare, contain content that pertains to the basic and cross-functional skills outlined in the O*NET. We provide theoretical, methodological, practical, and andragogical insights into the current state of training and learning content in online modalities. In doing so, the present study highlights whether a specific online learning platform is designed to effectively help individuals thrive in an evolving labor market.

### 5.1. Theoretical and Methodological Implications

Our study makes several theoretical and methodological contributions in the evolving field of work-relevant self-directed learning and reskilling. First, we investigate the learning ecosystem and identify the macro-factors (i.e., the availability of skill-relevant training) that can facilitate or impede work-relevant self-directed learning and development (Bronfenbrenner, 2009; Lyndgaard et al., 2024). We find evidence that existing online training programs provide opportunities to learn job-relevant skills, particularly those related to the modern world of work (e.g., bright outlook occupations). As such, this work draws attention to the learning ecosystem and highlights the potential importance of further consideration of assessment and opportunity in work-relevant learning (Beier et al., 2025). The availability of work-relevant skills training should affect whether workers and job seekers are spending their time, energy, and effort effectively to gain basic and cross-functional skills. Although our findings may seem intuitive, empirical evidence drawing from occupational and educational-related data to examine the extent to which online courses support the learning ecosystem for adult learners is scarce.

Second, we add to the growing body of literature using AI methodologies to examine talent development-related issues. The current study was designed as a proof of concept, such that in addition to our research questions, we wanted to understand the extent to which LLMs were effective at diagnosing which online courses prepare people in specific occupations, subsequently informing us about the opportunities people have to develop their talents. By situating the current study within the existing literature on AI in organizational science, we highlight innovative applications of AI that support the identification and assessment of online course content, which can facilitate workers and job seekers gaining basic and cross-functional skills. Further, our findings demonstrate how AI-driven analyses can effectively audit the talent development ecosystem for cognitive and emotional skill demands. For instance, we found robust coverage for cognitive skills (e.g., critical thinking; 81.5%) and socio-emotional skills (e.g., social perceptiveness; 27.9%), both of which are increasingly vital for holistic employee growth in the digital age.

### 5.2. Practical Implications

This study has several practical contributions. First, our work sheds light on the conversation around the mismatch between the predominant skills in the current workforce and the emerging skills required to remain competitive, otherwise known as the skills gap (Donovan et al., 2022). According to a recent McKinsey report surveying human resource professionals, 32% of employees lack the skills necessary to perform in their current role (Kirchherr et al., 2025). Although some argue that the skills gap exists because workers and job seekers do not possess the skills that are required for the job, another reason may be that employees and job seekers are not provided with adequate resources to gain the skills for specific job requirements. By demonstrating that online learning platforms are targeting basic and cross-functional skills, our work can help learners make better decisions about the online courses to engage in, subsequently increasing organizations’ agility (Jooss et al., 2024). Further, the specific platform used in our analysis, MIT OpenCourseWare, provides free online access to lecture notes, exams, assignments, and some video lectures, making it an accessible and cost-effective alternative for a wider audience of workers and job seekers unaffiliated with an organization (e.g., gig workers).

Second, although we did not directly study these issues, the use of course descriptions as content for research could inform andragogical studies. Course descriptions are the first point of contact with learners that communicate how courses contribute to learners’ academic and career aspirations (Lai et al., 2024). Indeed, the language included in course descriptions can impact enrollment intentions, course interest, expected workload, and learning outcomes (Mourey et al., 2021). Online course designers and instructors can examine the effect of course descriptions, particularly as related to the basic and cross-functional skills people can gain through engagement with the course. If learners are informed of the basic and cross-functional skills that translate to their current job role and job family, then they can make informed decisions on where to put their time, effort, and energy towards reskilling. Adult learners especially value detailed and clear course content and descriptions (Ausburn, 2004). As adult education theory suggests (e.g., andragogy; Knowles, 1980), clear and detailed course description can help adult learners find personal relevance in what they learn and create learning outcomes based on their real-world needs (e.g., reskilling).

Third, our findings suggest that reskilling programs should continue to target basic skills (e.g., mathematics, reading comprehension) that can be applied across jobs, sectors, or industries in addition to cross-functional skills (e.g., critical thinking, time management; Lee et al., 2025). Indeed, we found that each basic skill outlined by the O*NET could be acquired in courses on MIT OpenCourseWare. However, we acknowledge that several cross-functional skills, particularly those that are technical (e.g., troubleshooting, installation, repairing), are difficult to develop through an online modality. This is in part due to challenges of how to reliably assess hands-on abilities and provide constructive feedback in an online format (Mesuwini & Mokoena, 2024).

We also acknowledge that several socio-emotional skills are difficult to learn through online modalities. Although socio-emotional competencies appear in the MIT OpenCourseWare corpus, their coverage is concentrated in perceptual- and influence-oriented capabilities. For example, socio-emotional skills such as social perceptiveness, persuasion, service orientation, and instructing appear in 27.9%, 27%, 11.7%, and 5.3% of courses, respectively. This pattern contrasts with process-oriented skills such as active learning and critical thinking, which were linked to 88% and 81.5% of courses, respectively. Our course-skill linkages rely on binary judgments from course descriptions rather than observed learning activities. Thus, socio-emotional practice that occurs during delivery, such as peer critique or studio work, may be under-signaled, even when present. Practically, learners who aim to build socio-emotional skills, especially helping and teaching capabilities, may need to add practice-rich experiences such as structured peer coaching, client projects, or role play simulations to complement typical MIT OpenCourseWare offerings, while perceptual and influence skills can often be cultivated within existing course structures (Chernikova et al., 2020; De Haan & Nilsson, 2023).

Finally, the current study demonstrates the practical value of using an LLM-based approach. Technological advances in NLPs over the past decade have made it possible for researchers to apply LLMs to experimentation, measurement, and practice at scale (Demszky et al., 2023). Specifically, in practical scenarios where large-scale, general-purpose language modeling is necessary, LLMs are particularly well-suited (Abdurahman et al., 2024). In addition to the current study, researchers and practitioners in the field of employee training and development have increasingly applied LLM-based approaches to facilitate immersive and conversational learning experiences, wherein employees can practice skills in a controlled environment (Bernhardt et al., 2025). For instance, Violakis (2025) adopted an LLM-based approach to develop real-time personalized learning experiences that resembled real-life policing scenarios for police trainees and law enforcement personnel. We illustrate one valuable use case in which LLM-based approaches can enhance the experience of skill development and acquisition across a wide breadth of occupations.

## 6. Limitations and Future Research Directions

This work is not without limitations. First, due to fair use and web scraping policies, we only used one online learning platform, limiting the generalizability of our findings. Our work does not speak to the training-skills demands fit for all online learning platforms. It may be the case that other online learning platforms are better equipped to address the skills necessary for job families such as food preparation and serving or construction and extraction. Future research should expand the online learning platforms from which course descriptions are extracted (e.g., Coursera, LinkedIn Learning). Furthermore, future research should explore whether specific online learning platforms incrementally improve the proportion of online courses that cover important skills.

Second, inter-rater reliability among SME course-skill linkages was below the traditional threshold of 0.80 (i.e., Krippendorff’s Alpha (α) was 0.35; Artstein & Poesio, 2008; Hayes & Krippendorff, 2007; Krippendorff, 2011). The relatively low estimated Krippendorff’s Alpha for our human rater sample suggests that the rating task was highly subjective. In particular, the low magnitude reflects the inherent difficulty that human experts faced when attempting to infer abstract O*NET skills from brief, high-level course descriptions. This high variability among SMEs serves as a critical empirical justification for our LLM-based approach. Whereas human classification is subject to inconsistency and individual interpretation, the LLM provides a deterministic and scalable alternative that applies a unified logic consistently across the entire dataset.

Third, although we provide empirical justification for our LLM-based approach, LLMs have several risks associated with their use. For instance, hallucinations and model collapse (i.e., the failure of LLMs to differentiate between human- and machine-generated content) are well-known limitations of LLMs (Johnson & Hyland-Wood, 2024). The wide range of benefits offered by LLMs for talent development tasks (e.g., efficiency, cost savings, scalability) may be offset by ethical issues such as data privacy and bias in training data (Somavarapu & Sharma, 2025). As such, Burtsev et al. (2024) state, “LLMs’ uncanny ability to generate humanlike text outputs can easily lead us to ascribe to them capabilities that they do not possess” (p. 4). Thus, human oversight is standard and, in some cases, critical. Future research should continue to explore how maintaining humans-in-the-loop can complement and even offset the limitations of LLMs.

Fourth, the O*NET has received critiques related to the skills information in the database, such that skills information in the O*NET is not granular enough to advance effective reskilling efforts (Nieves et al., 2024). That is, there are concerns that the rate at which O*NET is updated may not accurately capture rapid employment trends or specific skills in demand. Increased granularity of skills information and data allows researchers to forecast emerging skill requirements (e.g., experience with LLMs) and their demand over time within various levels (e.g., region, companies; Chen et al., 2024). An example Frank et al. (2019) provides is for the skill of programming. They argue that programming is too broad and does not describe distinctions among programming languages relevant to specific occupations. Future research should explore the extent to which skill groups range in their current granularity (i.e., technical versus social skills), and whether increased precision of skill information is predictive of learning and development outcomes. Further, future research should incorporate databases that are dynamic and pull labor market analytics (e.g., Lightcast; Nieves et al., 2024) to compensate for missing or incomplete skill descriptions.

Fifth the current study does not include person-level data. Much theorizing from job and skills matching literature focuses on the alignment between the individual to jobs and skills. A macro-level approach limits our ability to account for micro-level characteristics of the person (e.g., educational attainment, prior work experience, existing knowledge, skills, and abilities) that are important for determining the extent to which individuals are well-suited to succeed in specific online courses. By adopting a macro-level approach, we are unable to discern whether individual-level experiences and abilities are positively linked to participation in a higher proportion of online courses where important skills can be acquired. Future research should examine whether a mismatch between individuals’ current skills and skills required for employment impacts reskilling intentions and whether other person-level factors (e.g., motivation) explain this relationship. Further, future research should develop a process that enables workers and job seekers to compare their existing skillsets with in-demand skills to identify which online resources may help their development.

Finally, because we do not have person-level data, the current study cannot speak to individual-level learning outcomes resulting from workers and job seekers engaging in online courses that are incongruent with basic and cross-functional skills over time. On the one hand, in the skills matching literature, it is argued that mismatched workers may actually benefit more from challenges presented in their work environment as a result of not possessing the required skills (Gao & Feng, 2024). On the other hand, if workers remain mismatched for prolonged periods of time, they will continuously encounter challenges and pressures that may be insurmountable (Büchel & Mertens, 2004). Future research should adopt longitudinal approaches to examine whether negative training-skills demands fit (i.e., prolonged incongruence between online course content and important skills) results in positive or negative work-relevant outcomes over time.

## 7. Conclusions

Now more than ever, workers and job seekers will have to engage in reskilling activities to remain employed and employable. The current study provides a macro-level understanding of how online learning content impacts skill development and acquisition. In doing so, we provide fresh insights by using LLMs to unpack the extent to which online course descriptions contain content that showcases the basic and cross-functional skills individuals can acquire. More generally, this work serves as an invitation for researchers, organizations, and online course developers to better understand how to best prepare workers and job seekers for skill demands and requirements of the future of work.

## Figures and Tables

**Table 1:** List of 35 O*NET basic and cross-functional skills and definitions.

Skill Group	Skill	Definition
Complex Problem Solving	Complex Problem Solving	Identifying complex problems and reviewing related information to develop and evaluate options and implement solutions.
Content	Active Listening	Giving full attention to what other people are saying, taking time to understand the points being made, asking questions as appropriate, and not interrupting at inappropriate times.
Content	Mathematics	Using mathematics to solve problems.
Content	Reading Comprehension	Understanding written sentences and paragraphs in work-related documents.
Content	Science	Using scientific rules and methods to solve problems.
Content	Speaking	Talking to others to convey information effectively.
Content	Writing	Communicating effectively in writing as appropriate for the needs of the audience.
Process	Active Learning	Understanding the implications of new information for both current and future problem-solving and decision-making.
Process	Critical Thinking	Using logic and reasoning to identify the strengths and weaknesses of alternative solutions, conclusions, or approaches to problems.
Process	Learning Strategies	Selecting and using training/instructional methods and procedures appropriate for the situation when learning or teaching new things.
Process	Monitoring	Monitoring/Assessing performance of yourself, other individuals, or organizations to make improvements or take corrective action.
Resource Management	Management of Financial Resources	Determining how money will be spent to get the work done, and accounting for these expenditures.
Resource Management	Management of Material Resources	Obtaining and seeing to the appropriate use of equipment, facilities, and materials needed to do certain work.
Resource Management	Management of Personnel Resources	Motivating, developing, and directing people as they work, identifying the best people for the job.
Resource Management	Time Management	Managing one’s own time and the time of others.
Social	Coordination	Adjusting actions in relation to others’ actions.
Social	Instructing	Teaching others how to do something.
Social	Negotiation	Bringing others together and trying to reconcile differences.
Social	Persuasion	Persuading others to change their minds or behavior.
Social	Service Orientation	Actively looking for ways to help people.
Social	Social Perceptiveness	Being aware of others’ reactions and understanding why they react as they do.
Systems	Judgment and Decision Making	Considering the relative costs and benefits of potential actions to choose the most appropriate one.
Systems	Systems Analysis	Determining how a system should work and how changes in conditions, operations, and the environment will affect outcomes.
Systems	Systems Evaluation	Identifying measures or indicators of system performance and the actions needed to improve or correct performance, relative to the goals of the system.
Technical	Equipment Maintenance	Performing routine maintenance on equipment and determining when and what kind of maintenance is needed.
Technical	Equipment Selection	Determining the kind of tools and equipment needed to do a job.
Technical	Installation	Installing equipment, machines, wiring, or programs to meet specifications.
Technical	Operation and Control	Controlling operations of equipment or systems.
Technical	Operations Analysis	Analyzing needs and product requirements to create a design.
Technical	Operations Monitoring	Watching gauges, dials, or other indicators to make sure a machine is working properly.
Technical	Programming	Writing computer programs for various purposes.
Technical	Quality Control Analysis	Conducting tests and inspections of products, services, or processes to evaluate quality or performance.
Technical	Repairing	Repairing machines or systems using the needed tools.
Technical	Technology Design	Generating or adapting equipment and technology to serve user needs.
Technical	Troubleshooting	Determining causes of operating errors and deciding what to do about it.

Note. Skill groups, skills, and definitions were mapped onto O*NET-SOC codes (occupations) and extracted from the O*NET 30.0 Database (National Center for O*NET Development, 2025g). Basic skills include content and process skill groups. Cross-functional skills include social, complex problem-solving, technical, systems, and resource management skill groups.

**Table 2:** Job families included in O*NET.

Job Family	Example Occupation
Architecture and Engineering	Engineers
Arts, Design, Entertainment, Sports, and Media	Photographers
Building and Grounds Cleaning and Maintenance	Grounds maintenance workers
Business and Financial Operations	Accountants and auditors
Community and Social Service	Mental health counselors
Construction and Extraction	Electricians
Educational Instruction and Library	Library technicians
Farming, Fishing, and Forestry	Animal breeders
Food Preparation and Serving Related	Chefs and head cooks
Healthcare Practitioners and Technical	Registered nurses
Healthcare Support	Personal care aides
Installation, Maintenance, and Repair	Locksmiths and safe repairers
Legal	Lawyers
Life, Physical, and Social Science	Economists
Management	Chief executives
Office and Administrative Support	Postal service mail carriers
Personal Care and Service	Childcare workers
Production	Gas plant operators
Protective Service	Firefighters
Sales and Related	Cashiers
Transportation and Material Moving	Flight attendants

Note. The job family “Military-Specific” was not included in our analyses.

**Table 3:** Confusion matrix of agreement between LLM and unanimous human rater consensus.

	**LLM Rating:** 0 = Skill Absent from Course	**LLM Rating:** 1 = Skill Present in Course
**Unanimous SME Consensus:** 0 = Skill Absent from Course	247	106
**Unanimous SME Consensus:** 1 = Skill Present in Course	8	37

Note. The top-left (247 true negatives) and bottom-right (37 true positives) cells represent instances of total agreement between the LLM and the human experts. The top-right (106 false positives) represents ratings where the LLM identified a linkage that the SME panel did not unanimously identify. The bottom-left (8 false negatives) cell represents ratings where the LLM failed to identify a skill that all three SMEs agreed were present. Counts represent 398 total course-skills pairs validated; two observations were excluded due to missingness.

**Table 4:** Number and percentage of courses from MIT OpenCourseWare linked to skills by skill category.

Social Skills		
Skill	Number of Courses	Percentage of Courses
Coordination	513	20.1%
Instructing	134	5.3%
Negotiation	546	21.4%
Persuasion	689	27.0%
Service Orientation	298	11.7%
Social Perceptiveness	711	27.9%
**Content Skills**		
**Skill**	**Number of Courses**	**Percentage of Courses**
Active Listening	793	31.1%
Mathematics	1977	77.6%
Reading Comprehension	1749	68.6%
Science	1746	68.5%
Speaking	831	32.6%
Writing	1453	57.0%
**Complex Problem-Solving Skills**		
**Skill**	**Number of Courses**	**Percentage of Courses**
Complex Problem-Solving	1710	67.1%
**Process Skills**		
**Skill**	**Number of Courses**	**Percentage of Courses**
Active Learning	2242	88.0%
Critical Thinking	2078	81.5%
Learning Strategies	880	34.5%
Monitoring	354	13.9%
**Resource Management Skills**		
**Skill**	**Number of Courses**	**Percentage of Courses**
Management of Financial Resources	659	25.9%
Management of Material Resources	541	21.2%
Management of Personal Resources	1141	44.8%
Time Management	455	17.9%
**Systems Skills**		
**Skill**	**Number of Courses**	**Percentage of Courses**
Judgment and Decision-Making	995	39.0%
Systems Analysis	1119	43.9%
Systems Evaluation	1437	56.4%
**Technical Skills**		
**Skill**	**Number of Courses**	**Percentage of Courses**
Equipment Maintenance	428	16.8%
Equipment Selection	328	12.9%
Installation	162	6.4%
Operations Analysis	1367	53.6%
Operation and Control	452	17.7%
Operations Monitoring	190	7.5%
Programming	1230	48.3%
Quality Control Analysis	321	12.6%
Repairing	411	16.1%
Technology Design	639	25.1%
Troubleshooting	402	15.8%

**Table 5:** Mean and standard deviation of courses from MIT OpenCourseWare linked to skills by job family.

Job Family	*n*	Mean Percentage of Online Courses Linked to Skills	Standard Deviation
Computer and Mathematical	31	0.45	0.04
Life, Physical, and Social Science	59	0.44	0.04
Architecture and Engineering	55	0.43	0.03
Legal	7	0.43	0.03
Business and Financial Operations	45	0.43	0.04
Arts, Design, Entertainment, Sports, and Media	38	0.42	0.04
Educational Instruction and Library	61	0.41	0.03
Office and Administrative Support	51	0.41	0.05
Healthcare Practitioners and Technical	82	0.41	0.03
Management	54	0.40	0.02
Community and Social Service	14	0.39	0.01
Protective Service	25	0.39	0.04
Sales and Related	20	0.38	0.04
Healthcare Support	19	0.38	0.05
Farming, Fishing, and Forestry	11	0.35	0.13
Personal Care and Service	29	0.34	0.06
Transportation and Material Moving	49	0.33	0.09
Construction and Extraction	59	0.33	0.10
Production	102	0.32	0.09
Installation, Maintenance, and Repair	50	0.31	0.05
Building and Grounds Cleaning and Maintenance	8	0.30	0.08
Food Preparation and Serving Related	15	0.30	0.07

Note. *n* represents the number of unique occupations analyzed within each job family.

**Table 6:** Proportion of online courses from MIT OpenCourseWare linked to skills regressed on job family.

Variable	Estimate	*SE*	*t*
Intercept	0.39 ***	0.01	33.04
Protective Service (Reference)	-	-	-
Architecture and Engineering	0.04 **	0.01	3.04
Arts, Design, Entertainment, Sports, and Media	0.03 *	0.02	1.98
Building and Grounds Cleaning and Maintenance	−0.09 ***	0.02	−3.71
Business and Financial Operations	0.04 *	0.01	2.53
Community and Social Service	0.00	0.02	0.15
Computer and Mathematical	0.06 ***	0.02	3.76
Construction and Extraction	−0.06 ***	0.01	−4.37
Educational Instruction and Library	0.02 †	0.01	1.66
Farming, Fishing, and Forestry	−0.04 †	0.02	−1.69
Food Preparation and Serving Related	−0.09 ***	0.02	−4.87
Healthcare Practitioners and Technical	0.02	0.01	1.42
Healthcare Support	−0.01	0.02	−0.37
Installation, Maintenance, and Repair	−0.08 ***	0.01	−5.52
Legal	0.04	0.03	1.51
Life, Physical, and Social Science	0.05 ***	0.01	3.36
Management	0.01	0.01	0.79
Office and Administrative Support	0.02	0.01	1.39
Personal Care and Service	−0.05 **	0.02	−3.09
Production	−0.07 ***	0.01	−5.45
Sales and Related	−0.00	0.02	−0.22
Transportation and Material Moving	−0.06 ***	0.01	−4.15

Note. *SE* = standard error. $R^{2}$ = 0.40. Adjusted $R^{2}$ = 0.39. *F*(21, 862) = 27.47, *p* < 0.001. † *p* < 0.10. * *p* < 0.05. ** *p* < 0.01. *** *p* < 0.001.

**Table 7:** Proportion of online courses from MIT OpenCourseWare linked to skills regressed on bright outlook occupation designation.

Variable	Estimate	*SE*	*t*
Intercept	0.39 ***	0.00	87.01
Bright Outlook Occupations (Reference)	-	-	-
Not Bright Outlook Occupations	−0.02 **	0.01	−3.26

Note. Two observations were deleted due to missingness. *SE* = standard error. $R^{2}$ = 0.01. Adjusted $R^{2}$ = 0.01. *F*(1, 880) = 10.61, *p* < 0.01. ** *p* < 0.01. *** *p* < 0.001.

## Data Availability

The data presented in this study are available in Massachusetts Institute of Technology: MIT OpenCourseWare at https://ocw.mit.edu/ (accessed on 15 October 2025). These data were derived from the following resources available in the public domain: National Center for O*NET Development and https://www.onetcenter.org/dataUpdates.html (accessed on 21 February 2026).

## Supplementary Materials

The following supporting information can be downloaded at: https://osf.io/eqxfk/overview?view_only=adb48666ad5c435ca0901f1956c087e9 (accessed on 21 March 2026).
